# High-throughput screen of essential gene modules in *Mycobacterium tuberculosis*: a bibliometric approach

**DOI:** 10.1186/1471-2334-13-227

**Published:** 2013-05-20

**Authors:** Guangyu Xu, Bin Liu, Fang Wang, Chengguo Wei, Ying Zhang, Jiyao Sheng, Guoqing Wang, Fan Li

**Affiliations:** 1 Key Laboratory of Zoonosis, Ministry of Education, Norman Bethune College of Medicine, Jilin University, Changchun, Jilin, China; 2 The First Bethune Hospital of Jilin University, Changchun, Jilin, China

**Keywords:** Mycobacterium tuberculosis, Essential gene modules, Operon, Pathway

## Abstract

**Background:**

Tuberculosis (TB) is an infectious disease caused by *Mycobacterium tuberculosis* (*M. tuberculosis*). The annotation of functional genome and signaling network in *M. tuberculosis* are still not systematic. Essential gene modules are a collection of functionally related essential genes in the same signaling or metabolic pathway. The determination of essential genes and essential gene modules at genomic level may be important for better understanding of the physiology and pathology of *M. tuberculosis*, and also helpful for the development of drugs against this pathogen. The establishment of genomic operon database (DOOR) and the annotation of gene pathways have felicitated the genomic analysis of the essential gene modules of *M. tuberculosis.*

**Method:**

Bibliometric approach has been used to perform a High-throughput screen for essential genes of *M. tuberculosis* strain H37Rv. Ant colony algorithm were used to identify the essential genes in other *M. tuberculosis* reference strains. Essential gene modules were analyzed by operon database DOOR. The pathways of essential genes were assessed by Biocarta, KEGG, NCI-PID, HumanCyc and Reactome. The function prediction of essential genes was analyzed by Pfam.

**Results:**

A total approximately 700 essential genes were identified in *M. tuberculosis* genome. 40% of operons are consisted of two or more essential genes. The essential genes were distributed in 92 pathways in *M. tuberculosis*. In function prediction, 61.79% of essential genes were categorized into virulence, intermediary metabolism/respiration,cell wall related and lipid metabolism, which are fundamental functions that exist in most bacteria species.

**Conclusion:**

We have identified the essential genes of *M. tuberculosis* using bibliometric approach at genomic level. The essential gene modules were further identified and analyzed.

## Introduction

Tuberculosis (TB) is an infectious disease caused by *Mycobacterium tuberculosis *(*M. tuberculosis*) [1,2]. In recent years, the prevention and treatment of TB have become difficult due to the prevalence of co-infection with HIV, drug resistance and uncertainty of Bacillus Calmette-Guérin (BCG) prevention [3-5]. Essential genes are those genes required for cell growth and survival [6,7]. Previous studies on the essential genes of *M. tuberculosis* pathogenesis primarily using gene knockout or RNA interference [8]. This approach is expensive and inefficient, and due to limitations of experimental techniques, no experimental method can achieve an essential gene screen at a High-throughput level [9,10]. Essential gene modules are a collection of functionally related essential genes in the same signaling or metabolic pathway [11]. The determination of essential genes and essential gene modules at genomic level may be important for better understanding of the physiology and pathology of *M. tuberculosis*, and also helpful for the development of drugs against this pathogen.

To date, more than 31 genomes of *Mycobacterium spp*. have been sequenced including nine *M. tuberculosis* strains [12]. However, the systematic analysis of functional genomics and metabolic regulation were not established in *M. tuberculosis*. In this study, we used a bibliometric approach and performed a High-throughput screening of five *M. tuberculosis* strains to identify the essential genes. We further analyzed the essential operons and pathways, based on early-established genomic operon database and annotation of gene locus [13-15].

## Material and methods

### Bibliometric method

The bibliometric was used as previously described [16]. The keywords “*Mycobacterium tuberculosis*” “H37Rv” “essential gene” have been used to search the publications from 2002 to 2011 in PubMed, MEDLINE, BiosisPreview, EMbase and SciFinder. Using Epidata3.1, the duplications of literatures and unrelated literatures were deleted by parallel entry and logical error test. A total of 819 literatures were retrieved and 112 literatures were used to analysis the essential gene modules.

### Ant colony algorithm

Multiple sequence alignment bases on BLAST algorithm was restricted by the length and number of the sequences. So, in this study, we used ant colony algorithm to optimize it [17,18]. First, we divided all sequences into several parts and gain an initial population of K as a column, N as a row. K is the number of individuals of the K-substituting groups; N is the number of sequences. d_ij_ indicates the i^th^ individual dividing positions on the j^th^ sequence. For the array, the calculation formula of *fitness(r)* is as follow:

$$\begin{array}{c} \mathrm{fitness}\left(r\right)=\sum_{i=1}^{N-1}\sum_{j=i+1}^{N}\mathrm{SE}\left(S_{i}^{p}\left(d_{\mathrm{ri}}\right),S_{j}^{p}\left(d_{\mathrm{ri}}\right)\right)\\ \;+\sum_{i=1}^{N-1}\sum_{j=i+1}^{N}\mathrm{SE}\left(S_{i}^{s}\left(d_{\mathrm{ri}}\right),S_{j}^{s}\left(d_{\mathrm{ri}}\right)\right) \end{array}$$

The following parameters were used for analysis: the Initial = 5, d_1_ = 2, d_2_ = 2, d_3_ = 3, NCmax (The maximum number of iterations) = 100, m(number of ants) = 100; Parameters for information volatile degree are p = 0.05, q = 0.03, q_1_ = 0.6, q_2_ = 0.35, q3 = 0.2, a = 5, b = 3, c = 2, T_1_ = 50 , T_2_ = 79 , T_3_ = 99, Q_1_ = 0.1, Q_2_ = 0.2.

## Results

### Operons and pathways of screened essential genes in *M. tuberculosis strains*

The genome of the highly pathogenic *M. tuberculosis* strain H37Rv has been sequenced [19]. Using bibliometric analysis, 684 essential genes were identified in H37Rv strain, 617 genes were proved by experiments as well as 67 genes were identified using an in *silico* approach. (Related literature of these genes listed in Additional file 1: Table S1). These genes were evenly distributed in the genome, consistent with the previous study [20]. The genomes of *M. tuberculosis* among the five strains (H37Rv, H37Ra, CDC1511, F11, and KZN1435) were highly conserved. Therefore, we searched the Gene Bank (http://www.ncbi.nlm.nih.gov/genbank) using ant colony algorithm to look for essential genes of other reference strains (H37Ra, CDC1511, F11, and KZN1435). The essential genes of these strains were 702, 665, 699 and 697, respectively (Table 1). It was worth noting that the number of essential genes in the different strains are varies, although the genomes of *M. tuberculosis* are highly conserved. This phenomenon is mainly caused by the total number of genes in each stain are different, and some essential gene will divide into two genes in another genome, which caused the differences of essential genes.

**Table 1:** Essential genes, operons and pathway in reference strains H37Rv, H37Ra, CDC1511, F11, KZN1435

		**Operons**		**Pathways**		
**Strains**	**Essential genes**	**Contain 1 essential gene**	**Contain more than 2 essential genes**	**Contain 1 essential gene**	**Contain less than 50 essential genes**	**Contain more than 50 essential genes**
H37Rv	684	185(60%)	122(40%)	7(8%)	82(89%)	3(3%)
H37Ra	702	192(59%)	134(41%)	7(8%)	82(89%)	3(3%)
CDC1551	665	178(60%)	119(40%)	7(8%)	82(89%)	3(3%)
F11	699	186(60%)	124(40%)	7(8%)	82(89%)	3(3%)
KZN1435	697	185(59%)	131(41%)	7(8%)	82(89%)	3(3%)

**Figure 1:**
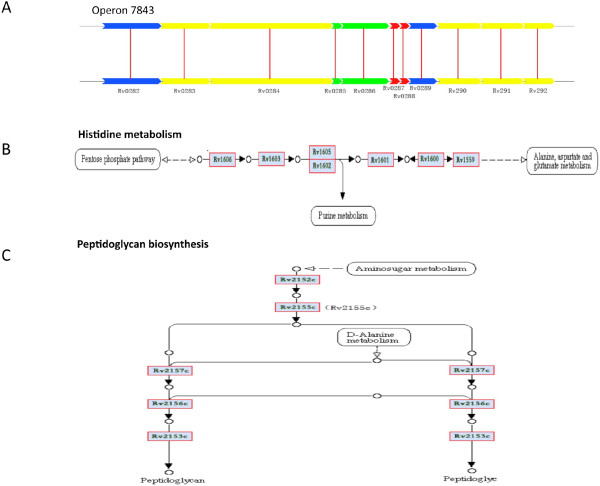
**Essential gene modules operons and pathways.** (**A**) An illustration for operon 7843. Non-essential genes are marked in red; PE/PPE family related genes are marked in green; possible conserved membrance genes are marked in yellow; hypothetical protein are marked in blue. (**B**) An illustration for histidine metabolism pathway. (**C**) An illustration for petidoglycan biosynthesis pathway.

We used operon database (DOOR) to assess the essential gene modules. These essential genes were fallen in 307, 326, 297, 310 and 316 operons in H37Rv, H37Ra, CDC1511, F11 and KZN1435, respectively (Table 1). Statistical analysis showed that there is no relationship between the size of the operon and the number of essential genes identified. In all strains, approximately 40% of operons are consisted of two or more essential genes and some operons controlled more than ten genes, suggesting that these operons may play an important role in physiology or pathogenesis. For example, the operon ID7843 controls eleven genes, nine of which are essential genes (Figure 1A). Of them, five genes are related to cell membrane, two genes encode protein related to PE/PPE family and another two genes encode hypothetical proteins.

Pathway is a signal transduction network that involves in multiple gene interaction. We analyzed the essential genes using pathway databases Biocarta, KEGG, NCI-PID, HumanCyc and Reactome. Although the numbers of essential genes in the five strains are slightly different, these essential genes have the same number of pathways (Table 1). The 684 essential genes of the H37Rv strain were distributed in 92 pathways. Of them, seven pathways only have one essential gene; 82 pathways have less than 50 essential genes; three pathways have more than 50 essential genes. It is interesting to note that in a portion of the pathway is entirely constituted by essential genes, which adjacent to each other in the genome. Histidine metabolism pathway, which is related to intermediary metabolism and respiratory, involves in ten essential genes. Seven of them are adjacent to each other and these clustered genes (*Rv1599-1606*) are required for L-histidine synthesis [21] (Figure 1B). Peptidoglycan synthesis pathway, which is related to cell wall and membrane formation, involves in ten essential genes (Figure 1C). Five essential genes (*Rv2152-2157*) are clustered together in the genome. Two of them are involved in N-acetyl muramic acid synthesis and the others for uridine monophosphate (UMP) synthesis [22]. We speculated that the linked genes are required for proper function and play crucial roles in pathways.

### Function prediction of essential genes in *M. tuberculosis*

We used Pfam (http://pfam.sanger.ac.uk/) to predict the potential function of the essential genes by analyzing the functional domains of encoded proteins. The functions of the essential genes are categorized into replication, regulatory proteins, virulence, intermediary metabolism and respiration, cell wall related, signal pathways, lipid metabolism, PE/PPE family, insertion sequences/phages and unknown (Table 2). 61.79% of essential genes are fallen into virulence, intermediary metabolism/respiration, cell wall related, signal pathways and lipid metabolism. 0.06% of essential genes are fallen into replication, regulatory proteins, PE/PPE family, and insertion sequences/phages; 38.15% of essential genes into unknown.

**Table 2:** Function predictions of essential genes for reference strains H37Rv, H37Ra, CDC1511, F11, KZN1435

	**H37Rv**	**H37Ra**	**CDC1551**	**F11**	**KZN1435**
Replication	10	10	10	10	10
	(0.01%)	(0.01%)	(0.02%)	(0.01%)	(0.01%)
Regulatory proteins	10	10	10	10	10
	(0.01%)	(0.01%)	(0.02%)	(0.01%)	(0.01%)
Virulence	288	293	284	292	291
	(42.10%)	(41.74%)	(42.71%)	(41.77%)	(41.75%)
Intermediary metabolism and respiration	265	270	260	269	266
	(38.74%)	(38.46%)	(39.10%)	(38.48%)	(38.16%)
Cell wall related	297	302	295	302	299
	(43.42%)	(43.021%)	(44.36%)	(43.20%)	(42.90%)
information pathway	251	255	248	256	253
	(36.70%)	(36.32%)	(37.29%)	(36.62%)	(36.30%)
Lipid metabolism	257	263	254	263	260
	(37.57%)	(37.46%)	(38.20%)	(37.63%)	(37.30%)
PE/PPE family	11	14	8	14	13
	(0.02%)	(0.02%)	(0.01%)	(0.02%)	(0.02%)
Insertion seqs and phages	12	13	10	13	12
	(0.02%)	(0.02%)	(0.02%)	(0.02%)	(0.02%)
Hypothetical/unknown	261	269	255	269	268
	(38.15%)	(38.31%)	(38.35%)	(38.48%)	(38.45%)

## Discussion

In the current study, we have done a High-throughput screen for essential genes of *M. tuberculosis*. A total approximately 700 essential genes are identified in the genome, some genes were proved by experiments as well as some genes were identified using an in *silico* approach. We further identified the operons and pathways of these essential genes and predicted the functions of these genes.

The numbers of essential genes in the different strains are distinct suggesting that although the genome of *M. tuberculosis* is highly conserved, variations exist among different strains. The differences lead to the various capacities of virulence, evolution, and immunogenic among *M. tuberculosis* strains. Therefore, the investigations on the difference among essential genes in different strains probably gain insight the new mechanism of pathogenesis, especially between the virulent stain (H37Rv) and avirulent stain (H37Ra).

In our study, there were about 40% operons having two or more essential genes. Some operons have as much as ten essential genes. In the pathway analysis, some pathways are consisted of as much as 50 essential genes. At present, there is no any experimental methods can perform the scanning of essential genes aspect for *M. tuberculosis*. In order to further verify whether these identified genes are essential genes, we used pathway analysis to found that if multiple essential genes are adjacent to each other and constitute known essential pathway, we highly suspected these genes identified are essential, which is critical for drug or vaccine development. Histidine metabolism pathway and peptidoglycan synthesis pathway were found in this study base on pathway enrichment analysis,most genes in these two pathways were essential genes and adjacent to each other. In this case, in-depth studies of above two pathways maybe provide more broad perspective for the new drug development.

Function analysis revealed that 61.79% of essential genes were categorized into virulence,intermediary metabolism/respiration,cell wall related and lipid metabolism, which are fundamental functions that exist in most bacteria species [23,24], however, insertion sequences, phages and horizontal transfer genes (HTG) are also founded. The function of insertion sequence in *Mycobacterium tuberculosis* are till obscure, and several literatures report that insertion sequences plays a vital role in the growth cycle, which are essential for the bacteria [25,26]. The PE/PPE family is *M. tuberculosis*-specific and is involved in *M. tuberculosis* infection and virulence. PE/PPE genes accounted for 10% of *M. tuberculois* genome. Several essential genes that are related to PE/PPE family were also identified in this study, which plays an important role in cell wall synthesis [27].

## Conclusion

In current study, we have identified the essential genes of *M. tuberculosis* using bibliometric approach at genomic level. The essential gene modules were further identified and analyzed.

## Abbreviations

DOOR: Database of prOkaryotic OpeRons; KEGG: Kyoto Encyclopedia of Genes and Genome; UMP: Uridine monophosphate

## Competing interests

The authors declare that they have no competing interests.

## Authors’ contributions

Conceived and design: GW, FL. Data collection: JS, FW. Analyzed the data: CW, BL, YZ. Wrote the paper: GX, GW. All authors read and approved the final manuscript.

## Pre-publication history

The pre-publication history for this paper can be accessed here:

http://www.biomedcentral.com/1471-2334/13/227/prepub

## Supplementary Material

Additional file 1: Table S1 All essential genes collected with Bioliometric approach. Click here for file
